# The effectiveness of cognitive-behavioural consultation on sexual function and sexual self-efficacy of women after childbirth

**DOI:** 10.1016/j.eurox.2022.100157

**Published:** 2022-07-06

**Authors:** Elham Erfanifar, Nasser behroozi, Seyed Mahmoud Latifi, Zahra Abbaspoor

**Affiliations:** a Department of Midwifery, Reproductive Health Promotion Research Center, Ahvaz Jundishapur University of Medical Sciences, Ahvaz, Iran; b Shahid Chamran University of Ahvaz, Ahvaz, Iran; c Diabetes Research Center, Health Research Institute, Department of Epidemiology & Biostatistics, School of Public Health, Ahvaz Jundishapur University of Medical Sciences, Ahvaz, Iran

**Keywords:** Cognitive-behavioral therapy, Sexual self-efficacy, Postpartum, Sexual dysfunction, Counseling

## Abstract

**Objective:**

The purpose of this study was to investigate the effect of the cognitive-behavioral therapy program (CBT) on the sexual function and sexual self-efficacy of 100 women after childbearing.

**Methods:**

In this experimental pretest-posttest and control group design study, women who passed eight weeks of their delivery and were referred to three health centers, in Shadegan, Iran, from January to April 2019 registered using the simple sampling method and randomly allocated into two groups of case and control. For the CBT group eight weeks of counseling (one session/week), and for the control group, routine educations were provided by health care providers. Both groups filled out the female sexual function index and sexual self-efficacy questionnaires before intervention and eight weeks after the last session.

**Results:**

There was no significant difference between the CBT and control groups before the intervention in terms of sexual function and sexual self-efficacy, but eight weeks after the intervention, there was a statistically significant difference between the two groups (P = 0.001).

**Conclusion:**

Proper implementation of counseling based on the CBT model improved sexual function and self-efficacy of nulliparous women after childbirth.

## Highlights

• Cognitive-behavioural consultation can improve sexual function of nulliparous women after childbirth.
• Cognitive-behavioural consultation can improve self-efficacy of nulliparous women after childbirth.
• Sexual desire is changed after birth, and sex education and counseling during this period are important.

## Introduction

Sexual health has an essential role at any stage of a women's life [1], [2] and sexual dysfunction could adversely affect the women’s quality of life of the postpartum women [3], [4]. Childbearing is a critical life transition that affects postpartum sexual function [5]. Following childbirth, a woman's sexual functioning may negatively have affected by Psychological distress and decreasing interest in resuming Sexual intercourse [5], [6]. Sexual dysfunction due to some factors such as personality, social, cultural, and family status may remain unknown or appear in the form of depression, low self-esteem, marital dissatisfaction, and separation [7], [8].

This disorder refers to trouble with sexual desire and psychosocial changes which disturb the sexual response cycle and lead to some conflicts in the couple^'^s relationship [9]. According to Rosen's theory, sexual function includes six components of sexual desire, sexual arousal, lubrication, orgasm, sexual satisfaction, and vaginal pain during and after intercourse[2].

The results of a meta-analysis of 59 studies have shown that if couples have sexual activity during pregnancy and enjoy it, their communication, gratitude, and behavior become more stable four months after childbirth and three years afterward [10]. Postpartum sexual dysfunction has been estimated to range from 25 % to 63 % [11]. Khajehei et al. also studied 325 women who had given birth a year earlier and reported sexual dysfunction in 3.64% and sexual dissatisfaction in 5.70%. Also, the most common sexual dysfunction was related to sexual desire (2.81 %), orgasm (3.53 %), and sexual arousal (3.52 %) [9]. In a study, 35 % of women reported a moderate level and 13 % of women reported a severe level of sexual dysfunction during 3 months after birth that improved over the time [12]. Studies show that inadequate sex education and information about sexual activity have a role in sexual dysfunction, incidence, and persistence of sexual disorders [13].

For the treatment of sexual disorders, several methods, including cognitive-behavioral therapy, yoga, medications, change in lifestyle, and psychiatric treatment, are applied [14], [15], [16], [17], [18], [19], [20]. Cognitive-behavioral therapy is a common method in the treatment of sexual dysfunction[18], [19], [20], sexual self-efficacy [21], depression and anxiety, and improving sexual satisfaction and marital relationship [22].

Because of the importance of sexual function after birth, and role of sexual relationships in the preservation of family, inadequate information on women regard to changes in sexual desire after birth, and also, the effect of sex education and counseling, especially the impact of CBT on the improving sexual function, this study designed to investigate the effectiveness of cognitive-behavioral consultation on sexual function and sexual self-efficacy of women after childbirth.

## Material and methods

This study was an experimental pretest, posttest control group design. Eighty -Four women, after eight weeks of delivery, entered the study. Women were referred to three health centers in Shadegan, Iran, between April and November 2019. Before data collection, written informed consent was obtained.

Inclusion criteria were: having basic literacy, being primiparous, availability during the next two months, having a normal vaginal delivery or cesarean section, and passing eight weeks of delivery. Exclusion criteria were included: chronic mental or physical illness, severe marital conflict, an adverse event such as child disease, being a drug or alcohol user, having a history of chronic disease, having sexual disorders in their partner, death of close relatives, having a history of abuse or sexual abuse before marriage and absence in more than two consecutive counseling sessions. The protocol of the present study was approved by the Ethical Committee of the Ahvaz Jundishapur University of Medical Sciences (Ref No: IR.AJUMS.REC.1398.687).

## Sampling method

The sample size was calculated by the following formula:

$$n=\frac{{\left(Z_{1-\alpha /2}+Z_{1-\beta }\right)}^{2}\;\left(S_{1}^{2}+S_{1}^{2}\right)}{{\left({\overset{®}{X}}_{1}-{\overset{®}{X}}_{1}\right)}^{2}}$$

$$\left\{\begin{array}{c} Z_{1-\alpha /2}=1.96\\ Z_{1-\beta }=0.84 \end{array}\right\}\left\{\begin{array}{c} S_{1}=1.21\\ S_{1}=1.77\\ {\overset{®}{X}}_{1}=6.63\\ {\overset{®}{X}}_{2}=5.06 \end{array}\right\}$$

According to Rostamkhani et al. study, the sample size was calculated at 42 for each group based on a test power of 80%, and a confidence coefficient of 95 %[23]. Considering an attrition risk of 20%, this number increases to 50 in each group.

## Measures

The qualified women were asked to fill out a demographic questionnaire, female sexual function index (FSFI), and Sexual self-efficacy questionnaire (SEQ). The researcher (EE) was present if women had any questions.

### Female sexual function index (FSFI)

The FSFI questionnaire has 19 questions and 6 domains of sexual activity including desire, arousal, lubrication, orgasm, pain, and sexual satisfaction. Two questions measure sexual desire, four questions measure lubrication and arousal domain, and three questions measure orgasm, sexual satisfaction, and pain domains. A five-point Likert scale was used for scoring. The total score of each domain was multiplied using a specific factor. The factor for desire was 0.6, for arousal and lubrication was 0.3, and for other domains was 0.4. Sexual dysfunction is defined as a total number of FSFI < 26 [2]. The validity and reliability of the FSFI questionnaire in Iran were evaluated by Fakhri et al. and indicated that this questionnaire is proper for measuring sexual function among Iranian women [24]. All women answered the Persian version of the FSFI questionnaire.

### Sexual self-efficacy questionnaire

This questionnaire was developed by Schwarzer, R., & Jerusalem, M. (1995). This questionnaire consists of 10 questions that are scored in four-choice categories, from 1 to 4 (Not at all true to exactly true). The total score is the sum of the scores of all items and it is between 10 and 40. A higher scores indicates higher self-efficacy. Internal reliability for general self-efficacy was Cronbach’s alphas between 0.76 and 0.90 and its validity was correlated to emotion, optimism, and work satisfaction. Negative coefficients were related to health complaints, stress, depression, anxiety, and burnout [25]. In preliminary research in Iran, the validity and reliability of the self-efficacy questionnaire have been confirmed by Vaziri et al. The reliability of this questionnaire was 0.86 using Cornbrash's alpha measurement method and its validity was confirmed by using the content validity method [26]. In the present study, using Cornbrash's alpha method, the reliability coefficient was calculated to be 0.83.

## Randomization

Eligible women after completing the informed consent form were randomly assigned into two CBT or control groups. Randomized blocking was used and a block size of 4 and an allocation ratio of 1:1 was considered. The pre-test was taken by the researcher.

## Intervention

Fifty women in the intervention group who gave birth were referred to the health centers after eight weeks of their normal vaginal delivery, and according to inclusion and exclusion criteria received eight counseling sessions (one session/week). The objectives of the study were explained to the participants and they were assured about confidentiality; also, informed consent (oral and written) were obtained from all the participants.

Women were visited by a psychologist during the first visit. The counseling sessions were held in 5 groups of 10, and each session lasted 90 min. The control group received only routine training and one compact disk on CBT, at the end of the intervention.

A summary of the content of the treatment sessions is given in Table 1.

**Table 1:** The educational content of meetings.

Session 1	In this session, women, introduced themselves to each other, and the researcher (EE) explained the sessions’ rules, sexual function and sexual self-efficacy, the cognitive-behavioral model, and homework.
Session 2	In this session, it explained how women can reduce their stress, according to the cognitive-behavioral model.
Session 3	Incorrect thoughts and attitudes about sexual function and sexual self-efficacy after childbirth and the relationship between negative thoughts and well-being were explained in the third session. The homework was reviewed.
Session 4	In this session, women were educated about the reconstruction and change of their unreasonable and negative attitudes and muscle relaxation. The homework was reviewed.
Session 5	Women were trained about strengthening positive self-talk, successful relationships, cognitive challenges, and increasing realism. The homework was reviewed.
Session 6	The researcher educated muscle relaxation and individually educated women about stress and its symptoms. The homework was reviewed.
Session 7	Self-practicing, changing negative thoughts, and also the effects of revised thinking were educated. The homework was reviewed.
Session 8	In the 8th session, women were educated about meditation, coping steps, practice, and the summary of all sessions. At the end of the intervention, the women in the control group were given one compact disk on CBT. The homework was reviewed.

## Statistical analyses

All data were entered in Chicago, Illinois: SPSS Inc. USA, version 23. For evaluating the normal distribution of data, the Kolmogorov-Smirnov test was used. The independent *t*‑test or Mann–Whitney test was used for comparing continuous data between the two groups, and Paired t-test was used to compare data within the groups. Also, for categorical data, the Chi‑square test was used. Analysis of covariance was performed to neutralize confounding effects. If the F value was significant, Toki tracking tests were used. The test power was 80%, and the level of significance was set at p < 0.05.

## Results

The mean age of participants was 22.54 (SD = 4.4) and 21.77 (SD = 4.0) in the CBT and control groups, respectively. There was no significant difference in education, economic situation, and type of childbirth between the two groups (Table 2).

**Table 2:** Socio-demographic Characteristics of Participants in CBT and control groups.

Group			CBT n = 42	Control n = 42	P-value
Variable					
**Women age**			22.54 ± 4.45	21.77 ± 4.08	0.20
**Women's education**	high school		7 (% 14.67)	8 (% 11.97)	0.27
	bachelor		28 (% 66.68)	30 (% 71.42)	0.28
	Master		15 (% 35.71)	12 (% 28.57)	0.30
**Spouse education**		high school	6 (% 11.42)	4 (% 9.52)	0.28
		bachelor	26 (% 64.76)	29 (% 69.04)	0.30
		Master	10 (% 23.80)	12 (% 28.57)	0.29
**Economic situation**		Low	15 (% 35.71)	11 (% 26.19)	0.18
		Average	25 (% 59.52)	27 (% 64.28)	0.28
		High	10 (% 23.80)	12 (% 28.57)	0.21
Mode of delivery		Cesarean Section	28 (% 66.66)	30 (% 71.42)	0.30
		Normal delivery	22 (% 52.38)	20 (% 47.67)	0.29

The mean score of sexual self-efficacy within the CBT group was improved after eight weeks of treatment compared to before treatment, 20.56 (SD = 5.2) and 10.43 (SD = 2.8) respectively (p = 0.001). Specifically, after the treatment, the mean score of Sexual self-efficacy in the CBT group was 20.56 (SD = 5.2) which was higher than that in the control group 10.55 (SD = 2.6), significantly (p = 0.001). Table 3 shows improving the sexual function domains over time (p < 0.001).

**Table 3:** Different domains of sexual function and sexual self-efficacy in CBT and control groups before and after intervention.

Variable		Before the intervention n = 42	After the intervention n = 42	P-value **
**Sexual self-efficacy**	CBT	10.43 ± 2.87	20.56 ± 5.22	0.001
	Control	10.13 ± 2.59	10.55 ± 2.66	0.578
P-value*		0.578	0.001	
**Desire**	CBT	2.11 ± 0.56	3.46 ± 0.83	0.001
	Control	2.20 ± 0.68	2.08 ± 0.70	0.532
P-value 1		0.577	0.001	
**Arousal**	CBT	2.55 ± 0.66	4.46 ± 0.97	0.001
	Control	2.76 ± 0.50	2.76 ± 0.50	0.598
P-value *		0.622	0.001	
Lubrication	CBT	2.18 ± 0.69	4.87 ± 0.97	0.001
	Control	2.33 ± 0.75	2.50 ± 0.80	0.588
P-value *		0.609	0.001	
**Orgasm**	CBT	2.00 ± 0.49	4.28 ± 0.89	0.001
	Control	1.97 ± 0.40	2.03 ± 0.50	0.503
P-value *		0.566	0.001	
dyspareunia	CBT	3.88 ± 0.79	1.89 ± 0.52	0.001
	Control	3.94 ± 0.50	3.13 ± 0.86	0.711
P-value *		0.755	0.001	
**Satisfaction**	CBT	2.08 ± 0.47	4.66 ± 0.90	0.001
	Control	2.11 ± 0.52	2.15 ± 0.58	0.400
P-value *		0.376	0.001	
**FSFI**	CBT	14.80 ± 3.66	23.69 ± 5.08	0.001
	Control	15.31 ± 3.35	14.10 ± 3.91	0.633
P-value 1		0.647	0.001	

* =p value between the two groups

* *=P value within the two groups

Table 4 present the results of the Tukey post hoc test on comparing sexual self-efficacy before and after interventions and also between the groups.

**Table 4:** Results of two-way analysis of variance related to sexual self-efficacy before and after the intervention.

Variable	Indices	Index	Value	F	P-value
**Sexual self-efficacy**	CBT	Pillais Trace	0.95	0.536	0.557
		Wilks Lambda	0.822		
		Hotelling's Trace	0.116		
		Roy's Largest Root	0.116		
	Pre-test * Post-test	Pillais Trace	0.521	13.557	0.003^**^
		Wilks Lambda	0.433		
		Hotelling's Trace	1.167		
		Roy's Largest Root	1.167		
	The interaction of the group, pre-test, and post-test	Pillais Trace	0.600	8.657	0.004^**^
		Wilks Lambda	0.478		
		Hotelling's Trace	1.133		
		Roy's Largest Root	1.133		

**Fig. 1:**
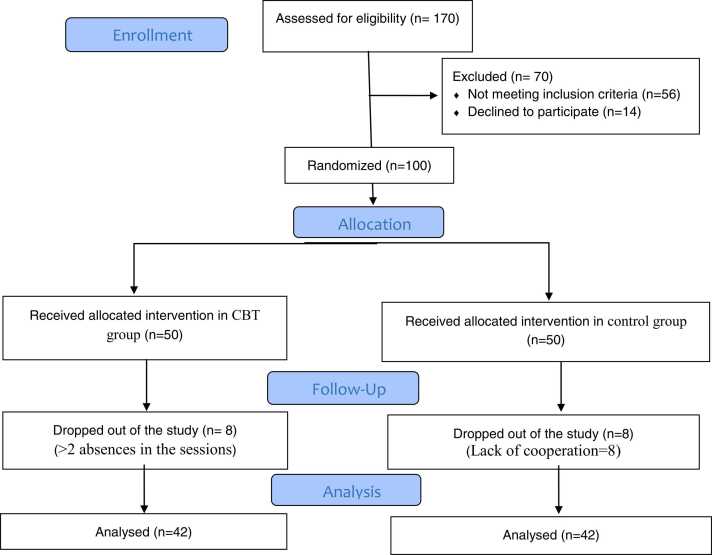
**:** Flowchart showing recruitment of participants into the study.

In the CBT group, eight women were excluded in the first week of the study, because of absence in ˃2 of the sessions. Also, eight women were excluded in the first week because of a lack of cooperation in the control group (Fig. 1).

## Discussion

This study investigated the effectiveness of cognitive-behavioral therapy on sexual function and self-efficacy in primiparous women after childbirth. Our findings demonstrate a significant improvement in postpartum sexual self-efficacy and sexual function after CBT sessions.

In the CBT group, the sexual function of the women in the phases of sexual desire, arousal, orgasm, lubrication, dyspareunia, and satisfaction was improved considerably. In consistent with the results of the present study, Rostamkhani et al. study showed that PLISSIT-based counseling compared to routine counseling increased the sexual function scores significantly [25]. Also, in another study mindfulness, and cognitive-behavioral therapy improved sexual desire, arousal, and satisfaction [27]. Hamid et al. conducted a semi-experimental with a pretest, posttest plan, and follow-up with a control group. The results showed that cognitive-behavioral counseling reduces anxiety and fear of intimacy in women with vaginismus just after treatment sessions and after a 12-month follow-up period [25].

The other finding of this study was that the cognitive-behavioral counseling compared to the routine model increases the scores of sexual self-efficacy after childbirth, so that in the test group before the intervention, 100% of women had a poor sexual self-efficacy score. But after the eight counseling sessions, there was an increasing trend in boosting postpartum women's sexual self-efficacy.

Ismaeilzadeh et al. reported that cognitive-behavioral therapy could improve mental health and increases women's sexual self-efficacy [28]. Besides, cognitive-behavioral therapy could have affected the lifestyle and sexual self-efficacy and promoted the women's psychological health [29], marital self-efficacy, and marital satisfaction [30]. These results are in line with our results.

Nezamnia et al., in their randomized controlled trial study, found that cognitive-behavioral therapy during pregnancy increases performance, sexual satisfaction, and self-efficacy [31]. Based on the findings, is concluded that cognitive-behavioral counseling is effective on primiparous women's sexual function and sexual self-efficacy after childbirth by reducing stress and anxiety and improving stress coping strategies.

## Conclusion

This study found that CBT can improve sexual self-efficacy and sexual function in all aspects of it (Desire, Arousal, Lubrication, Orgasm, Dyspareunia, and Satisfaction) in primiparous, postpartum women. In postpartum counseling centers for women with sexual dysfunction, the CBT model can be a useful way to treat this disorder, and health care providers can learn and apply this method to improve sexual function and life satisfaction of postpartum women. However more research with more extended follow-up is suggested.

## The study’s strengths and limitations

This is the first research that investigated the effects of CBT on sexual function and self-efficacy among postpartum women in Iran. However, there are some limitations to this study. First, recall bias can influence responses to sexual function and self-efficacy questionnaires. Second, this study was not blinded, although the researchers and the women did not know who was in which group until the sampling began**.**

## CRediT authorship contribution statement

The conceptualization and data collection were undertaken by E.E as the first author. N.B, as the second author contributed to the methodology and supervision of counseling sessions, and SM.L contributed to the formal analysis of data, software, and validation. Z.A., as the fourth author contributed to the methodology and supervision in all stages of study, writing, review, and editing of the article. All authors agree with the final version of the manuscript to be submitted to the journal, and they met the criteria of authorship.

## Declaration of Competing Interest

The authors declare that there is no conflict of interest.
